# Giving Voice to the Medically Under-Served: A Qualitative Co-Production Approach to Explore Patient Medicine Experiences and Improve Services to Marginalized Communities

**DOI:** 10.3390/pharmacy6010013

**Published:** 2018-01-27

**Authors:** Asam Latif, Sana Tariq, Nasa Abbasi, Baguiasri Mandane

**Affiliations:** 1 School of Health Sciences, University of Nottingham, Nottingham NG7 2RD, UK; 2 Karimia Ltd., Nottingham NG7 5JU, UK; sana@radiodawn.com; 3 Queen’s Pharmacy Centre, Nottingham NG8 5DB, UK; queenspharmacycentre@hotmail.com; 4 University Hospitals of Leicester NHS Trust, Leicester LE1 5WW, UK; baguiasri.mandane@uhl-tr.nhs.uk

**Keywords:** adherence, co-production, e-learning, medically under-served, medicines use review (MUR), pharmacy

## Abstract

**Background:** With an aging population, the appropriate, effective and safe use of medicines is a global health priority. However, “‘medically under-served” patients continue to experience significant inequalities around access to healthcare services. **Aim:** This study forms part of a wider project to co-develop and evaluate a digital educational intervention for community pharmacy. The aim of this paper is to explore the medicine needs of patients from marginalized communities and suggest practical way on how services could be better tailored to their requirements. **Method:** Following ethical approval, qualitative data was gathered from: (1) workshops with patients and professionals (*n* = 57 attendees); and (2) qualitative semi-structured interviews (10 patients and 10 pharmacists). **Results:** Our findings revealed that patients from marginalized communities reported poor management of their medical conditions and significant problems with adherence to prescribed medicines. Their experience of pharmacy services was found to be variable with many experiencing discrimination or disadvantage as a result of their status. **Discussion:** This study highlights the plight of medically under-served communities and the need for policy makers to tailor services to an individual’s needs and circumstances. Furthermore, patients and professionals can work in collaboration using a co-production approach to develop educational interventions for pharmacy service improvements.

## 1. Introduction 

With an aging population, the appropriate, effective and safe use of medicines is a global health policy priority [1]. Despite medicines prescribing being the most common patient-level healthcare intervention, adherence to medicines for long-term conditions is estimated to be 25–50% [2,3,4]. It is well recognized that patients require support to improve adherence and address medicine-related problems such as concerns over dependence, tolerance and side effects [5,6,7]. Medicine non-adherence leads to a substantial worsening of disease and associated increased rates of hospitalization [8,9]. It also has an economic impact with one estimate suggesting the annual cost of medicines wastage to the National Health Service (NHS) is £300 million; including £90 million that is retained in individuals’ homes and £110 million returned to pharmacies [10]. Globally, policy-makers are increasingly turning to community pharmacy to develop and fund medication review services [11]. Pharmacies are well placed within the community, and their extended opening hours means they are more accessible than other professionals [12]. In addition, the pharmacy profession has ambitions to broaden the pharmacists’ roles towards cognitive pharmaceutical services (defined by Cipolle et al. as the use of specialized knowledge by the pharmacist for the patient or health professionals for the purpose of promoting effective and safe drug therapy [13] and management of chronic illness that will extend its remit beyond the predominantly medicine supply function [14]. 

In England, one commissioned medication review service known as the “Medicines Use Review” (MUR), has been available from community pharmacies since 2005 [15]. An MUR involves an annual face-to-face patient–pharmacist consultation that seeks to improve the patient’s knowledge of prescribed medicines and their use (Box 1). Pharmacies are currently entitled to claim a fee of £28 for each MUR they undertake with patients and can conduct up to maximum of 400 reviews annually.

Box 1 **The purpose of the MUR service.**

The MUR aims, with the patient’s agreement, to improve his or her knowledge and use of drugs. This purpose is to be achieved through:

1. Establishing the patient’s actual use, understanding of and experience of taking his or her medications;
2. Identifying, discussing and resolving poor or ineffective use of medicines by the patient;
3. Identifying side effects and drug interactions that may affect patient compliance and
4. Improving the clinical and cost effectiveness of prescribed medicines thereby reducing the wastage of such drugs [15].

The value of MURs to patients is not conclusive. Research studies demonstrating the most efficacy have been those targeting patients with specific medical conditions such as asthma [16] and cardiovascular disease [17]. However, there have been concerns expressed over completeness, legibility of MUR forms and disparity in the recommendations made to GPs [18,19]. Furthermore, early research into the implementation of MURs raised questions over their value to patients and whether they are being targeted to “*local needs and patient priorities*” [20]. In 2011, commissioners responded to these concerns with reforms to improve the service, stipulating the targeting of MURs to patients with specific medical needs, as research indicated these patients might benefit to a greater degree [21]. These were identified as those who: 1. are taking medicines associated with hospital admissions (NSAIDs, anticoagulants, antiplatelets, diuretics); 2. are discharged from hospital with medicine changes; and 3. have respiratory disease. Under these new terms, at least 50% of all MURs undertaken by the pharmacy should be with patients in these categories. In 2015, the proportion was raised to 70% and a fourth target group was introduced (patients at risk of or diagnosed with cardiovascular disease and regularly being prescribed at least four medicines) [21].

The changes to service delivery reflect ongoing objectives to target the service to those who could benefit the most. However, the reforms have not sought to target patients who are “hard-to-reach” or medically under-served. This is despite mounting evidence suggesting that patients belonging to these communities (e.g., people with disabilities, people from Black, Asian and Minority Ethnic (BAME) backgrounds, the home-bound, homeless, Gypsy, Roma and Traveller communities) typically experience poorer health outcomes than the general population and find it more challenging accessing health and screening services [22,23,24,25]. The problem for policy makers is that there is a low level of evidence to support medication reviews in these groups as they are generally under-represented in research. There is therefore a lack of research evidence to support reforms to target these groups (i.e., there is little evidence to suggest people belonging to a medically under-served community would benefit any more from an MUR than those who are not [26,27]. Furthermore, any reforms to target and orientate service remuneration towards these patients may also be complicated due to ambiguity over who should be classed as being medically under-served, and the practicalities of how they could be identified and approached within the community pharmacy setting. Nevertheless, research continues to highlight that patients who are medically under-served have poorer inequitable access to health care due to them experiencing greater physical barriers to accessibility, encountering poorer patient-professional communication and are significantly disadvantaged where a service is not tailored to their unique needs or preferences [28]. 

The aim of this paper is to explore the medicine taking experiences and needs of patients from marginalized communities, and suggest practical way on how services could be better tailored to their requirements. A qualitative co-production approach [29] was used in order to collect evidence to inform a digital e-learning resource to be used as an educational intervention for pharmacy staff. 

## 2. Method

Four mixed patient-professional workshops and twenty qualitative one-to-one interviews were held in autumn and winter 2016. A broad range of patients identified as being medically under-served and a range of professional pharmacy stakeholders were invited to either a workshop or interview according to their preference. Medically under-served patients were recruited via local self-help groups, and national health and social care websites (e.g., Healthwatch England). “Pharmacy teams” were sampled purposefully and recruited through local and national pharmacy representative websites. Full details are available from the research protocol, which has been published elsewhere [26].

### 2.1. Access 

Following a review of the literature and building on the New Medicines Service (NMS) evaluation study [30], a list of patients identified as belonging to a medically under-served community was initially compiled. This was reviewed by participants during workshops. The list was not designed to be exhaustive but aimed to illustrate a range of people, who by virtue of their circumstances, could be medically under-served and may require and benefit from additional support with medicines from the pharmacy (Box 2).

Box 2 **List of people who could be medically under-served.**

- People with disability i.e., people with physical disability (e.g., a person in a wheelchair); people with visual impairment (Partially sighted/blind); people with hearing impairments (deaf) people with learning impairment (e.g., Downs syndrome, autism etc.)
- People who are housebound
- People from Gypsy, Roma and Traveller communities
- People who are homeless or have no fixed address
- People who are refugees or seeking asylum
- People from the lesbian, gay, bisexual and transgender, queer (LGBTQ) communities
- People from Black, Asian and Minority Ethnic (BAME) communities
- People with mental health illness and stigmatised medical conditions (e.g., acquired immune deficiency syndrome (AIDS), epilepsy)
- Older people, particularly with multiple morbidities and medicines
- Young people (specifically men aged 18–25)
- People from rural communities (e.g., people located outside towns and cities)
- People from a low socio-economic status (UK National Statistics Socioeconomic classification) or low levels of health literacy defined as the “*personal characteristics and social resources needed for individuals and communities to access, understand, appraise and use information and services to make decisions about health*” [31]
- People with speech disorders (e.g., stutter) or language disorders e.g., from brain injury (stroke, dementia)
- People with alcohol/drug dependency
- People who have experienced domestic/physical abuse
- Women who visit the pharmacy with small children
- People who are long-term unemployed
- People who are sex workers
- People in prison or those who are known to have been in prison

A patient sampling frame was then developed and patients (or an organizational representative) were then recruited via local community support organizations. Recruitment was facilitated by advertisements in the East Midlands Academic Health Science Network (AHSN, Nottingham, UK) “Public Face” newsletter. A range of pharmacy professionals was also purposefully recruited including pharmacy staff directly involved with the day-to-day recruiting of patients for an MUR. 

### 2.2. Co-Development and Review Woskshops 

Two mixed patient/pharmacy staff “development” workshops were arranged at the University of Nottingham and led by Latif to discuss: patient stories of belonging to a medically under-served community, experiences of how they managed their medicines, pharmacy use and knowledge of MURs, and strategies that pharmacy staff could use to further engage them and the communities. Working together in small groups to share knowledge and explore ideas, both patients and pharmacy teams provided detailed written accounts of their personal experiences. The focus was for patients to report their medicine problems and reflect on instances how belonging to an under-served community could constrain engagement and access to healthcare. The workshop participants were also asked to develop an outline for the contents of the e-learning. They were also asked to capture thoughts on the design aspects of the e-learning such as illustrations and preferred media. Personal written reports along with group suggestions to improve the MUR service were captured on “storyboards” to further elicit a more detailed sketch of the contents of the e-learning. 

Following the development workshops, two “review” workshops followed which further refined the ideas into a detailed written specification (e.g., working title, learning objectives, contents of topics to be covered). The e-learning development team then used this information to create the resource. A £200 inconvenience allowance was made available to all workshop participants whether they were patients or pharmacy staff. The amount of money offered reflected an average day’s pharmacist locum fee.

### 2.3. Qualitiave Interviews 

One-to-one, face-to-face qualitative semi-structured interviews were undertaken by Latif with medically under-served patients and pharmacy professionals to further inform the e-learning intervention. Interviews were approximately 30–45 minutes and with the participants’ consent, audio-recorded. Patient interviews focused on understanding patient medicine taking experiences, their use of pharmacy use and thoughts on how pharmacy staff can better engage with and improve services for the medically under-served (Box 3). These interviews were also necessary to ensure participants who were unwilling or unable to attend the workshop (i.e., they were housebound) were represented.

Box 3 **Abridged patient topic guide.**

**Background**

- Explore background e.g., work, lifestyle etc.
- Explore health status, medical conditions and current concerns about health
- Use of health services
- Investigate experiences of health care system
- Advice/support offered by the GP or other health professionals
- Explore concerns or problems with medications
- Explore adherence i.e., are medicine taken as prescribed or have these been changed? Miss doses, if so when/why?
- Explore understanding of what medicines are for, concerns about side effects, perceptions of effectiveness, reluctance to take etc.

**Relationship with pharmacy/pharmacist**

- Frequency of use of local pharmacy
- Experiences with pharmacies and support staff
- Have patients been offered a pharmacy consultation, if so explore context
- Patient awareness of available pharmacy support and MUR service
- Feelings about being approached for support services i.e., MURs
  Specific questions relating to belonging to an under-served community
- Explore obstacles to access
- Explore communication/cultural challenges
- Difficulties experienced by those with disability (People with disability)
- Reluctance to seek healthcare advice
- Participants’ views on how MURs can be better tailored to people who are medically under-served

**Developing the e-learning intervention**

- Explore what participant would like to see in a training resource for pharmacists/support staff to improve the care to the medically under-served

Final comments/thoughts

Pharmacy staff interviews sought to explore the current context in which the MUR was being delivered, awareness and engagement with the medically under-served and suggestions on what should be included in a digital educational intervention to improve engagement (Box 4).

Box 4 **Abridged pharmacy staff topic guide.**

**Background and current delivery of MURs**

- Explore work context/involvement in MURs/identification of patients for MUR
- What participants want to achieve from undertaking the service? Are there any people who are avoided?

**Awareness of the medically under-served**

- Who do participants believe would most benefit from an MUR
- Explore participants’ understanding of under-served/or “hard-to-reach” groups
- Previous experiences/frequency of undertaking MURs with patients from an under-served community
- Barriers to identifying and approaching patients
- Learning needs
- Explore pharmacy’s role and how well they cater to the medically under-served
- Previous training undertaken
- Explore how confident participants are engaging with the medically under-served
- Explore what should be included in an educational resource to improve service delivery

Final comments/thoughts

### 2.4. Co-Production Approach

The co-production concept is broad and can range from service co-planning and co-commissioning, service co-design and co-delivery, to co-assessment, co-monitoring and co-evaluation [29]. The co-production process was used in the development of the digital educational intervention (e-learning) for community pharmacy staff [26]. These themes that emerged from the data formed the basis for the e-learning intervention. The co-production approach served as an opportunity for pharmacy professionals to review how they offer services to the medically under-served. This reflective element sought to extend the understanding of the problems patients have managing medicines, how they cope and the support they feel was available to them [32]. It also adds to the limited evidence base suggesting vulnerable groups of patients face significant challenges utilizing health care services [33] and builds on previous co-production studies that seek to tailor health services to the unique needs and preferences of service-users [34,35].

Workshops and one-to-one interviews were used to explore medicine issues. Qualitative data was gathered to highlight the unique problems faced by a range of medically under-served patients, the challenges they experienced when accessing and using the health care system, and their relationship and use of pharmacy. The e-learning was therefore developed inductively and was sensitive to the issues of under-served communities. It sought to expose the medicine needs of patients from marginalized communities and suggest practical way on how services could be better tailored to their requirements. We provide details of the e-learning that was produced and how this can be accessed freely online (See Supplementary Materials).

### 2.5. Analysis 

Qualitative data analysis coincided with the initial stages of data collection. The analysis proceeded iteratively so that emergent findings from the initial workshop and interviews were built upon and incorporated into subsequent workshops and interview topic guides. All workshop accounts, ideas and notes from storyboards were narrated and transcribed. Audio-recorded interviews were transcribed verbatim. Data was then imported into the qualitative analysis package NVivo (Version 11) for coding through constant comparison. Latif independently coded the data with all authors sharing in the analysis process comparing interpretations to clarify the internal consistency of codes in order to identify conceptual relationships. Our focus was on understanding and unpacking the experiences of people belonging to an under-served community and how they managed their health and medicines. 

### 2.6. Ethical Approval 

Ethical approval was received from East Midlands Research Ethics Committee (REC reference: Derby 16/EM/0237) on 15 July 2015, along with governance clearance through the NHS Health Research Authority (HRA) (Nottingham, UK). There were several ethical challenges working with patients from medically under-served communities. During workshops, an “appreciative” approach was used throughout to promote inclusivity, agency and build trust. To reduce the potential for any “power differential” between patients and pharmacy professionals, it was made clear that the purpose of the workshop was to learn from patients’ expertise and their lived-experiences. 

## 3. Results

**Recruitment**

Twenty-nine participants (19 patients or organizational representatives, and 10 pharmacy professionals) attended the e-learning development workshops and 28 participants (14 patients or organizational representatives, and 14 pharmacy professionals) attended the review workshops. To support and further the findings from the workshops, twenty one-to-one interviews were also conducted with 10 medically under-served patients (5 male and 5 female; mean age 53). Patient representation was from: the asylum and refugee community; the BAME community including people whose first language is not English; people with disabilities; people who are homeless, people who are housebound, people from the lesbian, gay, bisexual and transgender and queer (LGBTQ) community; people diagnosed with mental illness; people from the Gypsy, Roma and Traveller (GRT) communities and older people with multiple morbidity and medicines. Ten Pharmacy staff also participated (7 female and 3 male; mean age 44) with the following job roles: community pharmacist, academic pharmacist, representative from the profession of pharmacy, Accredited Checking Technician (ACT), dispenser and a Medicines Counter Assistant (MCA).

**Findings**

Three themes emerged from the data, namely: 1. Personal circumstances and medicines; 2. Patients and the pharmacy; and 3. Tailoring MURs to the medically under-served.

### 3.1. Personal Circumstances and Medicines

Most patients’ accounts revealed unique personal challenges belonging to an under-served community. They revealed that their status often left them feeling socially isolated. They reported a lack of support networks, and several accounts revealed overwhelming and often tragic circumstances. Amid this, patients experienced significant barriers to accessing routine care:

I sleep rough, and have drug and alcohol dependency. I have a chaotic lifestyle. I am a sex worker and have suffered from domestic and sexual abuse, and was trafficked … I have asthma, deep vein thrombosis, schizophrenia [etc.]… I’m not taking any of my medications as I don’t have any money to get them dispensed … I don’t know who to trust. I think pharmacists work for the government. They ask funny questions. I will just use the hospital if I get sick.

*[Workshop: Female 30 yrs homeless]*

Most patients described poorly managed medical conditions and significant problems with medicine adherence. Non-adherence ranged from using the medicine inappropriately, adjusting medicine doses because of concerns over side effects or dependency, and stopping the medicine altogether. Patients’ lack of knowledge about medicines and their side effects meant they relied on their own experience and other sources of information to make informed decisions:

I came off myself because I didn’t feel well taking them. … I came off the blood pressure tablets as I can be poorly up to 3 weeks. It causes me pain taking the medicines. For cholesterol tablets, they were showing they had side effects on the TV news. With this confirmed I need to stop taking the tablets.

*[Workshop: Female 68 yrs Disability]*

For others adherence fluctuated according to their circumstances, and in complex cases this was due to more deep-rooted psychological issues:

I’m not very good at taking things … and that relates partly to my mental health … I almost see it as a subtle form of self-harming that I neglect my medications … If I was in a very bad place I wouldn’t take anything. It would depend on how I was feeling.

*[Interview: Female 66 yrs Multiple morbidities and medicines] *

Problems with medicines were also reported that were more specific to an individual community. For example, participants from the GRT community revealed that they often bought medicines from other patients as there was a strong taboo attending the doctor or pharmacy. It was found that issues were compounded where individuals belonged to more than one under-served community i.e., those who were homebound and had English as a second language experienced barriers both in their inability to physically access help as well as barriers to communication. In most cases patients typically relied on ad hoc assistance from whomever this could be sought:

I live on my own and English is not my first language. I am housebound and I am isolated … I get my prescriptions automatically so a lot of medicines are wasted especially the GTN sprays ... I’ve just been given a new medication for stomach cramps prescribed by the hospital. I’ve got this dispensed at the pharmacy … I’ve just been asking people who visit me how to take them and to read the information leaflet. I don’t speak English so I can’t call the pharmacy to explain.

*[Workshop: Female 62 yrs housebound]*

During workshops, pharmacy professionals were surprised and at times shocked at how challenging it was for patients to manage medicines. They were taken aback by patient reports of their experience and use of pharmacy:

I was surprised to hear the number of patients turning to illegal suppliers of medicines due to the poor experience of health care and pharmacy professionals

*[Workshop: Female 39yrs Pharmacist]*

I was surprised about the lack of entitled benefits. The lack of support available to the homeless, refugees, and sex workers … how they find the pharmacy to be unapproachable.

*[Workshop: Female 33 yrs Dispenser] *

Pharmacists acknowledged that patients from under-served communities were not receiving parity of care and that they needed to do more in order to facilitate access and build trust. There were indications that they were self-reflecting on the care they personally provided:

I have reflected that maybe I am not doing a good job because most of these groups are not getting the same level of care as the general public

*[Workshop: Male 29 yrs Pharmacist]*

I feel more enlightened after having spoken to patients. Patients feel frustrated and it has helped me understand the need to support her since she is struggling dealing with her condition.

*[Workshop: Female 32 yrs Pharmacist]*

### 3.2. Patients and the Pharmacy

When asked specifically about their experiences of using the pharmacy, patients’ opinions were mixed. Some conveyed gratitude and good opinion, and for individuals who had interacted with the pharmacist, they found this to be a friendly and a useful encounter. Others however reported poorer relationships and limited interactions. Pharmacies were generally perceived as busy places and this limited the scope for patients to raise issues with medicines:

I use my pharmacy every two months. They are very formal, have a corporate style, no interaction. I have never had a conversation with how I’m getting on. I’m continuing to use emollients but don’t feel this is working. I have cholesterol checks with the GP, but I don’t feel I need this. I’ve not had counseling; it is very rushed. There is a lack of space and long queues.

*[Workshop: Male 39 yrs BAME community]*

Patients centered their concerns on issues of communication and discrimination. Patients sometimes felt that the pharmacists were discouraged, knowing or unknowingly, from engaging with those whom it was more challenging to communicate e.g., where patients’ second language was not English. In these cases patients also felt embarrassed to initiate conversation, which closed off avenues for them to seek help and assistance:

Language is the main problem for my community and the pharmacies struggle a lot to cater for this, but at the same time it is not their fault as we are the people who cannot speak English … The pharmacist can only do what they can do under these circumstances.

*[Workshop: Male 72 yrs Refugee] *

If you as a pharmacist think they are not going to understand, so I don’t need to do a review … and the patient also thinks “oh I’m an old person, I’m from Asian background, I don’t know the language, so if I start asking questions they will be in trouble”, so they are discouraged. So in terms of language it works two ways.

*[Interview: Male 40 BAME]*

Communication concerns also extended to people with disabilities. For example patients from the deaf community described pharmacy interactions where they simply were unable to comprehend what was being said. Other than written instructions, which themselves could be confusing, they reported not receiving medicines information at the point of dispensing because pharmacists lacked the skills to sign and did not have access to an interpreter. Like in other cases, patients turned to their own community or used other sources (i.e., the Internet) to find out necessary information. The lack of engagement with the deaf community left patients feeling frustrated as they were having to make all the effort which was not always seen to be reciprocal:

I think deaf people now have become so secluded that they don’t bother anymore, and that’s made them so lonely and isolated … The pharmacist will find it’s not going to be smooth talking to this person as they won’t be able to communicate, so they will just grab all the things and give it to them … there is no communication, there is nothing

*[Interview: Female age not given Disability]*

Another emergent theme was the feeling of being judged or disadvantaged. A range of personal accounts of discrimination was reported both in the wider societal context but also when in the pharmacy, which made patients feel marginalized, isolated and neglected. For example, several participants belonging to the BAME community felt that they did not received the same care as others:

The pharmacy don’t offer these services to Asian people. They think it’s for non-Asians … I don’t know about MURs and the benefit they can have.

*[Workshop: Female 23 yrs BAME community] *

I have encountered discrimination due to wearing a headscarf where the pharmacist assumed I could not speak English ...

*[Workshop: Female 32 yrs BAME community]*

Another example reflects the experience of a patient who was homeless. They occasionally noticed a lack of engagement and good etiquette, which created the impression that they did not feel welcome. In cases involving asylum seekers, patients did not feel they could order medicines where these were needed, fearing they would be questioned about this. They did not feel they were entitled to the same level of rights to health services as others despite being eligible to receive the same levels of health care:

The relationship with the pharmacy is sometimes friendly and sometimes discriminative. Sometimes they are impatient with me. I feel very frightened most of the time … When I attend for my methadone I usually feel desperate. I want to talk but they seem very busy … I usually need to leave very quickly for lots of reasons. They never say I can come back if I am worried about anything, even when I look very ill.

*[Workshop: Female 30 yrs homeless]*

You are so severely dismissed when you are an asylum seeker. I feel dehumanized on a day-to-day basis because I don’t have the legal status. Patients don’t understand or have weak understanding of their rights, so have a weak understanding of medicines and how to use them.

*[Workshop: Male 43 yrs asylum seeker] *

Further examples of ways in which marginalized communities felt discriminated against came from the patients from the LGBTQ community. For example, those who were transgender felt anxious and judged because they felt they were being treated differently. The traveller community who also expressed views of discrimination:

People treat you as a third class citizen. People look down on you, they treat as like you’re weird … I’m a professional, I’ve got a degree, but people treat me like you’re a freak show.

*[Workshop: Non-binary 45 yrs Transgender] *

I would describe the relationship with the pharmacy as discriminatory. The community will not engage with the pharmacy because of a lack of trust. Pharmacists are usually ok, non-judgmental, but the pharmacy girls come across as judgmental and prejudiced … We have been part of this country for over 1000 years and still made to feel like invaders … It’s difficult to go to a pharmacy when you know you’re not welcome.

*[Workshop: Male no age given GRT community]*

Despite the large number of cases where discrimination was reported, there were instances where good practice and collaboration between the GP and the pharmacist had ensured that the patient received the appropriate support for managing their medicines:

I would have struggled without the help of my GP who asked my interpreter to assist me at the chemist after my one-to-one consultation with the GP. I do not need any further support

*[Workshop: Female 63 yrs Refugee] *

### 3.3. Tailoring MURs to the Medically Under-Served

With the exception of one patient, none of the patients in this study had been offered or received an MUR. Patients revealed that they would welcome an MUR if they were approached with dignity and respect. They preferred this to be undertaken during less busy periods in order for the pharmacist to build up a rapport and have time to listen to them “*unconditionally*”. Participants were asked about ways pharmacies could tailor their service to enable and empower patients to accept the offer. Several suggestions were made including the need to raise awareness of the service as it was felt many patients had poor awareness of MURs or knowledge about its availability. Participants were keen for pharmacists to be proactive and engage with patients who are medically under-served:

They should proactively engage with hard-to-reach people, because these are the people who need it most. It’s very strange that the people who need it most are getting the least. Because of the structure, attitude of people and other things going on

*[Interview: Male 40 yrs BAME]*

Patients were keen for pharmacy teams to undertake cultural competence training to seek a greater awareness and better understand the community they served:

If the community isn’t coming to you, then you have to go to the community. So there’s something about being proactive, about going to places where communities meet. Giving a little talk with some freebies maybe … I think that piggybacking on a social event for that community

*[Interview: Female 66 yrs Multiple morbidities and medicines]*

It was also suggested that pharmacists should not feel frightened to approach and enquire about a person’s circumstance if they thought this may affect medicine adherence:

They should recognize where people have an obvious difficulty in accessing materials. For example, blind people reading information sheets … The best thing to engage is to communicate … We need the help, but sometimes are not confident enough to ask for it or know who to ask

*[Workshop: Male 25 yrs disability]*

In circumstances where the pharmacist was unable to speak the patients’ native language, patients suggested a range of solutions including speaking slowly, taking longer with patients so they do not feel rushed, using hand gestures and taking time to explain how to use the medicine in a language they could understand. Where there was illiteracy, pictorial leaflets were suggested. It was recommended that the pharmacy should recruit staff that were representative of the community it serves (described as “ethnic matching”) and that the images on display posters should include more diversity. 

On hearing patient accounts and suggestions, pharmacy staff revealed that it was challenging in a busy retail environment to be able to offer a bespoke service. Barriers were varied and included concerns over how individuals would be identified, practical concerns over communicating with patient who did not speak English, and concerns over problematic access to the consultation area:

Various languages are spoken, Polish, Urdu, Romani and it is difficult to counsel appropriately let al.one do a full MUR. Additionally, access is an issue, high-risk patients tend to be housebound and therefore do not have access to services, and they aren’t offered these through the community pharmacy.

*[Workshop: Female 27 yrs Pharmacist] *

I think there are some I would avoid overtly. Especially people I have difficulty getting into the consultation room, so either some physical disabilities … so anybody in a wheelchair would have been out as I wouldn’t have been able to get them in.

*[Interview: Female no age given Pharmacist]*

Pharmacist reported that work and organizational pressures dehumanized service delivery making it challenging to offer a patient-centered service:

You do lose that ability of care, and you feel it, you all know that inside, but you don’t say anything. To those above it’s a target and something they want you to achieve … It’s not about giving that quality care

*[Interview: Female 58 Non-Pharmacist Manager]*

I think over the years what’s happened is nationally it’s almost like everything has to be the same, which then doesn’t work because it doesn’t accommodate all the little variations … So we need to go back and in each individual pharmacy, gear it towards the population that it is meant to be meeting the needs of [Interview: Female 38 yrs Pharmacist]

## 4. Discussion

Our findings show that people who are medically under-served may be experiencing significant health and medicine challenges that are going unnoticed. This supports a growing body of work that suggests patients from marginalized communities experiencing greater physical barriers to accessibility and encounter poorer patient-professional engagement [28]. It also suggests that that some patient groups may be particularly prone to being socially isolated and so experience a lack access to health care support. We also revealed that patient barriers to effective health care tended to be incremental, in that if patients belonged to more than one medically under-served group their barriers became compounded. Many pharmacy professionals were surprised at the low levels of care experienced by these patients but also acknowledged the barriers to practice change. 

There has been increased emphasis on the importance of co-designing health care services and how this process can be used to re-conceptualize the role of the patients at all stages of quality improvement process [36]. This approach seeks to facilitate a partnership between health professionals and service users to improve the patients’ experience [37,38]. Results from this study highlight how patient-professional collaboration can help bring the patient’s voice to the fore and offer suggestion on how service providers can tailor services meet patients’ needs and preferences. The strength of this approach is that equity is sought “first-hand” from the target audience and so contributing to a “bottom-up” approach. However, several limitations to this study are acknowledged. Most importantly, co-production depends on a subject or challenge being understood by all participants. It was found that most of the patient participants had not been offered or received an MUR. It is, therefore, not known to what extent patients were able to contextualize a service that they were not familiar with or understand how the e-learning would eventually be applied in practice. There was no reason to suspect the integrity of accounts, however, it is unknown to which these findings can be extended and representative of wider populations. During workshops, it is also not known what impact or influence the presence of pharmacy staff had on patients. Although an appreciative approached was taken, patients could have felt inhibited or worried about the comments they made in the presence of pharmacy staff. In addition, it is unknown to what extent the individual accounts can be effectively extrapolated into an educational e-learning format [39]. 

There are several important implications for policy makers, health professionals and educationalists. Firstly, is the lack of consideration given to service delivery when policies are translated into practice. Despite calls to make pharmacy services patient-centered [40] and to promote shared decision-making [41], significant progress is still needed to ensure all patients are offered equitable access to care. The policy adjustments made in 2011 stipulating the targeting of MURs to patients with specific medical needs [21], may need further revision to include patients groups from medically under-served communities to promote equitable access to the MUR service. Secondly, organizations need to review their current infrastructure and invest in training and development (i.e., cultural competence) so that front-line staff are both enabled and have the skills to build trust and respond to patients’ unique circumstance. Thirdly, there is a strong need for monitoring and review of equity of care, addressing unconscious biases and eliminating discrimination. Lastly, the e-learning that has been produced as a result of this study should be widely publicized in order for practice change to occur. Future research should seek to evaluate the impact and effectiveness of co-design approaches at improving the patients’ experience, health utility and health resource use.

### Digital Educational Intervention 

The findings presented in this paper contributed towards the final co-produced digital educational intervention. This comprised of three web-based resources namely:

1. Discovering and understanding under-served communities
2. Exploring the medicine experiences and needs of patients who are under-served
3. Effectively interacting and engaging patients who are under-served

The first resource reflected the low level of awareness among pharmacy professionals of the medically under-served as uncovered during workshop discussions and interviews. The second resource sought to develop empathy through understand the lived experiences of patients who are medically under-served. The last resource, provided the leaner with tips on how to enable patients to empower themselves to take up the offer of a Medicines Use Review. In order to engage the learner in interactive learning, each resource consisted of a mixture of multimedia elements (i.e., audio, images, activities and illustrative videos) and represents approximately 15–20 min of learning activity. The resource is freely accessible online and can be accessed here: (http://www.nottingham.ac.uk/helmopen/rlos/pharmacy/mur-compendium/).

## 5. Conclusions

This study highlights the significant problems patients from medically under-served backgrounds experience with medicines. Our findings suggest that there is a need for a pharmacy educational intervention to raise awareness of marginalized communities. A co-production approach was useful to develop the educational intervention and was a feasible method to promote a patient-centered agenda.

## Supplementary Materials

The co-produced e-learning resource can be accessed freely online at http://www.nottingham.ac.uk/helmopen/rlos/pharmacy/mur-compendium/.
